# A New Lignan Glucoside from the Whole Plants of *Salvia Scapiformis*

**DOI:** 10.3390/molecules180911377

**Published:** 2013-09-13

**Authors:** Yongji Lai, Shuiping Ding, Huiqin Qian, Jinwen Zhang, Yongbo Xue, Zengwei Luo, Guangmin Yao, Yonghui Zhang

**Affiliations:** 1 Hubei Key Laboratory of Natural Medicinal Chemistry and Resource Evaluation, School of Pharmacy, Tongji Medical College, Huazhong University of Science and Technology, Wuhan 430030, Hubei, China; E-Mails: laiyongji1024@163.com (Y.L.); qianhuiqina@163.com (H.Q.); bobbyschro@126.com (Y.X.); luozengwei@gmail.com (Z.L.); 2 Tongji Hospital Affiliated to Tongji Medical College, Huazhong University of Science and Technology, Wuhan 430030, Hubei, China; E-Mails: 13907113263@139.com (S.D.); tjzhangjinwen@163.com (J.Z.)

**Keywords:** *Salvia scapiformis*, lignans, triterpenoids, hydrolysis

## Abstract

Phytochemical investigation of the whole plants of *Salvia scapiformis* Hance afforded eight compounds, including one new lignan, (+)-8*α*-hydroxypinoresinol-8-*O*-[6′′-*O*-(4′′′-hydroxybenzoyl)]-*β*-d-glucopyranoside (**1**), four known lignans, (+)-8*α*-hydroxy-pinoresinol-8-*O*-*β*-d-glucopyranoside (**2**), (+)-8*α*-hydroxypinoresinol (**3**), (+)-pinoresinol (**4**), and (+)-medioresinol (**5**), and three known triterpenoids, ursolic acid (**6**), 4-*epi*-niga-ichigoside F1 (**7**), and niga-ichigoside F1 (**8**). Their structures were determined on the basis of extensive spectroscopic analyses and comparison with spectroscopic data in the literature. The absolute configuration of the new compound **1** was determined by chemical transformation and GC analysis.

## 1. Introduction

**Figure 1:**
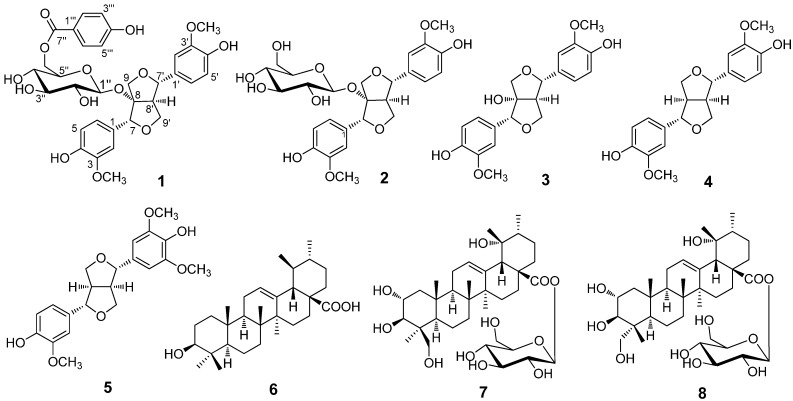
Chemical structures of compounds **1**–**8** from the whole plants of *Salvia scapiformis*.

*Salvia* is an important genus in the family Lamiaceae, 84 species of which are widely distributed all over the country in China [1]. Many species of this genus have been cultivated worldwide for ornamental and culinary purposes, and some species are used as folk medicine. *Salvia scapiformis* Hance, known as “Bai-Bu-Yao”, is used as a folk medicine in China to treat cough, hemoptysis, traumatic injuries, traumatic hemorrhage, dysentery, and furunculosis [2]. As part of our ongoing search for novel natural compounds from Chinese medicinal plants, phytochemical study on the Me_2_CO extracts of *S. scapiformis* resulted in eight compounds. In this paper, we describe the isolation and structure elucidation of one new lignan glucoside (**1**) and seven known compounds **2**–**8** (Figure 1).

## 2. Results and Discussion

The acetone extract of the whole plants of *S. scapiformis* was subjected to silica gel column chromatography (CC) to give seven fractions A–G. Further separation of fractions D and G provided compounds **1**–**8** (Figure 1). The known compounds **2**–**8** were identified as (+)-8*α*-hydroxypinoresinol-8-*O*-*β*-d-glucopyranoside (**2**) [3,4], (+)-8*α*-hydroxypinoresinol (**3**) [3], (+)-pinoresinol (**4**) [5], (+)-medioresinol (**5**) [6], ursolic acid (**6**) [7], 4-*epi*-niga-ichigoside F1 (**7**) [8], and niga-ichigoside F1 (**8**) [9], respectively, by comparison of their spectroscopic data with those reported in the corresponding literature.

**Figure 2:**
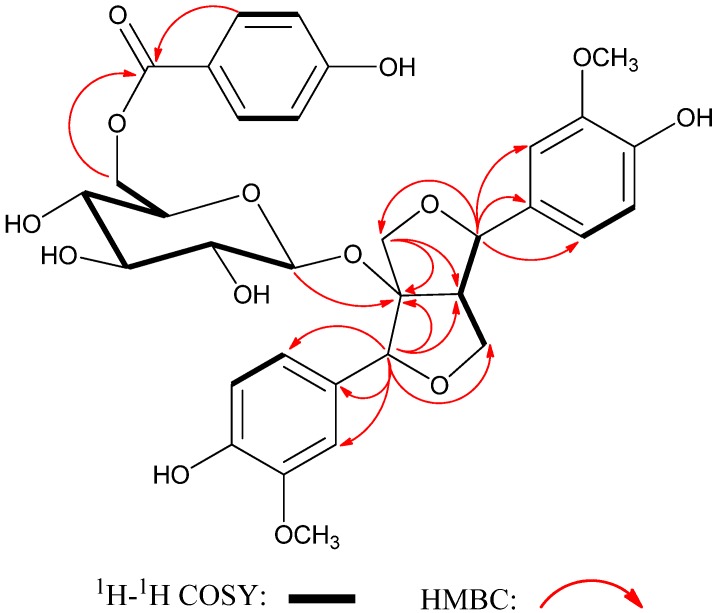
Key ^1^H-^1^H COSY and HMBC correlations of compound **1**.

Compound **1** was obtained as a colorless gum with the molecular formula C_33_H_36_O_14_, as deduced from HRESIMS at *m/z* 679.1985 [M + Na]^+^ (calcd for C_33_H_36_O_14_Na, 679.2003). The IR spectrum of **1** showed absorptions of hydroxy (3,359 cm^−1^), carbonyl (1,700 cm^−1^), and aromatic (1,607 and 1,517 cm^−1^) functionalities. In the ^1^H-NMR spectrum of **1**, two sets of 1,3,4-trisubstituted and one set of typical AA′BB′ coupling aromatic ring signals at *δ*_H_ 6.72 (1H, d, *J* = 8.1 Hz), 6.77 (1H, d, *J* = 8.1Hz), 6.81−6.85 (4H, overlapped), 7.01 (1H, d, *J* = 1.7 Hz), 7.03 (1H, d, J = 1.7 Hz), and 7.88 (2H, d, *J* = 8.8 Hz), two pairs of oxygen-bearing methylene protons at *δ*_H_ 4.46 (1H, t, *J* = 8.7), 4.38 (1H, d, *J* = 10.4), 3.85 (1H, d, *J* = 10.4), and 3.76 (1H, dd, *J* = 9.2 and 5.7 Hz), and two oxygen-bearing methyl singlets at 3.84 and 3.82 were observed. Its ^13^C-NMR spectrum showed two oxygenated methyl, three methylene, eighteen methine (ten aromatic and seven oxygenated), eight aromatic quaternary carbons, one oxygenated quaternary carbon, and one ester carbonyl. In addition, a series of signals in the range of about the protons at *δ*_H_ 3.0–4.5 and their corresponding carbons at *δ*_C_ 65.2, 72.0, 74.9, 75.7, 78.3, and 100.2 indicated a hexosyl sugar moiety [4]. In the HMBC spectrum, the correlations of H-2′′′/H-6′′′ (*δ*_H_ 7.88) and H-3′′′/H-5′′′ (*δ*_H_ 6.83) with C-1′′′ (*δ*_C_ 122.3), C-4′′′ (*δ*_C_ 163.8), and C-7′′′(*δ*_C_ 167.9) showed a *p*-hydroxybenzoyl unit (Figure 2). On comparison of its spectroscopic data with those of know lignans [2,3,4,5,6], compound **1** appeared to be a lignan derivant with a furofuran skelon. A long-range correlation from the anomeric proton H-1′′ (*δ*_H_ 4.38) to C-8 (*δ*_C_ 99.3) in the HMBC spectrum implied that the sugar unit was located at C-8 of aglycone. Moreover, the HMBC correlations between H-6′′ (*δ*_H_ 4.20 and 4.52) and C-7′′′ implied that the *O*-(*p*-hydroxybenzoyl) group was attached to C-6′′.

**Figure 3:**
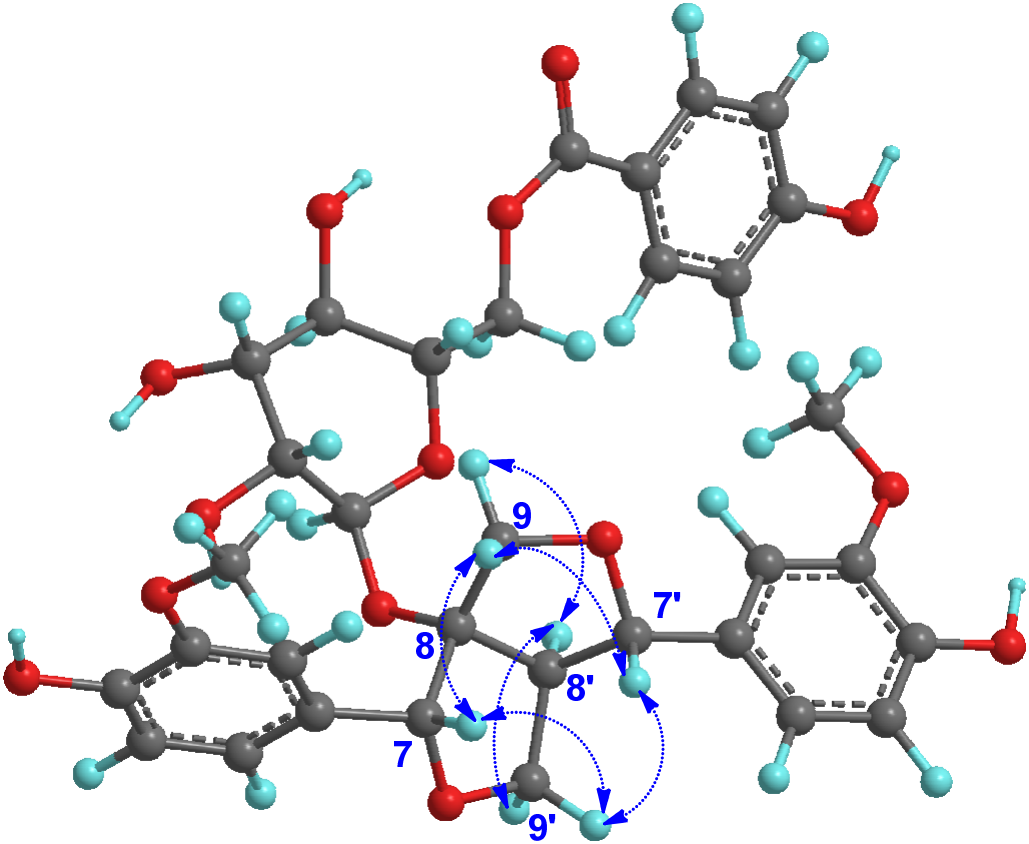
Key NOESY correlations of the furofuran skeleton of compound **1**.

The relative configuration of the furofuran skelon was determined by NOESY experiment (Figure 3). Assuming that H-8′ was *α*-oriented due to the known lignans (compounds **2**–**5**) from this plant [2,3,4,5,6], the *α*-orientations of H-9*α* (*δ*_H_ 4.38) and H-9′*α* (*δ*_H_ 4.46) were established from the NOESY correlations from H-8′ (*δ*_H_ 3.40) to H-9*α* and H-9′*α*. The NOESY correlations of H-9*β* (*δ*_H_ 3.85) and H-9′*β* (*δ*_H_ 3.85) with H-7 (*δ*_H_ 4.66) and H-7′ (*δ*_H_ 4.77) implied that H-7 and H-7′ should be positioned on the *β* face of the molecule.

Moreover, the structure and absolute configuration of compound **1** was further determined by chemical transformation. Alkaline hydrolysis of compound **1** gave *p*-hydroxybenzoic acid and compound **2**, and the later was hydrolyzed by cellulase (EC 3.2.1.4) to obtain compound **3** and sugar moiety. In addition, the sugar moiety was unambiguously determined to be *β*-d-glucose according to the coupling constant of its anomeric proton (*J* = 7.7 Hz) and GC analysis. Finally, compound **1** was elucidated as (+)-8*α*-hydroxypinoresinol-8-*O*-[6′′-*O*-(4′′′-hydroxybenzoyl)]-*β*-d-glucopyranoside.

## 3. Experimental

### 3.1. General

Optical rotations were measured on a Perkin-Elmer PE-341LC polarimeter. UV spectra were recorded using a Varian Cary 50 spectrophotometer. IR spectra were recorded on a Bruker Vertex 70 FT-IR spectrophotometer. NMR spectra were recorded on a Bruker AM-400 spectrometer, using the residual CD_3_OD (*δ*_H_ 3.31/*δ*_C_ 49.0) signals as references. HRESIMS data were acquired using an API QSTAR Pulsar spectrometer. Column chromatography was performed using silica gel (100−200 mesh and 200−300 mesh, Qingdao Marine Chemical Inc., Qingdao, China), Amberchrom CG161M (75 μm, Rohm and Haas, Philadelphia, PA, USA), ODS (50 μm, YMC, Kyoto, Japan), and Sephadex LH-20 (Pharmacia Biotech AB, Uppsala, Sweden). Semipreparative HPLC was performed on an Agilent 1100 liquid chromatography with an YMC (10 × 250 mm, 5 μm) column. GC analysis was performed with an Agilent Technologies 6890N gas chromatography system. Solvents were distilled prior to use, and spectroscopic grade solvents were used. TLC was carried out on precoated silica gel GF_254_ plates.

### 3.2. Plant Material

Whole plants of *S. scapiformis* Hance were collected at Enshi of Hubei Province, China, in September 2010, and authenticated by Dr. Jianping Wang of Huazhong University of Technology and Science. A voucher specimen (No. 2010-0902) was deposited in the herbarium of Hubei Key Laboratory of Natural Medicinal Chemistry and Resource Evaluation, Tongji Medical College, Huazhong University of Technology and Science.

### 3.3. Extraction and Isolation

The air-dried and powdered whole plants of *S. scapiformis* (27 Kg) was extracted with Me_2_CO (3 × 150 L) at room temperature for seven days. The extract was concentrated under the reduced pressure. The residue (1,300 g) was subjected to silica gel (100−200 mesh) column chromatography (CC) eluting with a gradient of petroleum ether–Me_2_CO–MeOH (1:0:0→0:1:0→0:0:1) to give seven fractions (A−G). Fractions D and G were decolorized on Amberchrom GC161M CC (eluted with 70%→100% EtOH−H_2_O) and subjected to ODS eluting with a gradient of MeOH–H_2_O (4:6→1:0) to obtain non-triterpene subfractions D′ and E′ and triterpene subfractions D′′ and E′′, respectively. Subfraction D′ (8.5 g) was further isolated over silica gel (125g) CC eluting with CHCl_3_–Me_2_CO (1:0→10:1) to provide four subfractions (D′_1_−D′_4_). Subfraction D′_2_ was purified over Sephadex LH-20 eluted with MeOH to give fractions D′_2.1_, D′_2.2_, D′_2.3_, and D′_2.4_. Subfraction D′_2.3_ was subjected to silica gel CC again, and compounds **4** (6 mg, t_R_ 28 min) and **5** (8 mg, t_R_ 32 min) were purified by semi-preparative HPLC eluted with MeOH–H_2_O (46:54, flow rate: 2 mL/min). Subfraction D′_2.4_ was fractionated successively by silica gel CC, Sephadex LH-20 eluted with MeOH, silica gel CC, and semi-preparative HPLC eluted with MeOH–H_2_O (35:65, flow rate: 2 mL/min) to give compound **3** (9 mg, t_R_ 25 min). One main compound was observed in subfraction D′′ by TLC, and compound **6** (40 g) was obtained by recrystallization in MeOH. Fraction G′ was subjected to repeated chromatography and purified by semipreparative HPLC to afford **1** (15 mg, t_R_ 26 min, MeOH–H_2_O = 45:55, flow rate: 2 mL/min) and **2** (8 mg, t_R_ 23 min, MeOH–H_2_O = 4:6, flow rate: 1.8 mL/min). Compound **7** (5 mg, t_R_ 26 min) was separated by semipreparative HPLC eluted with MeOH–H_2_O (6:4, flow rate: 1.5 mL/min) from subfraction E′′, and compound **8** (40 mg) was recrystallized from the same part.

*(+)-8α-Hydroxypinoresinol-8-O-[6′′-O-(4′′′-hydroxybenzoyl)]-β-D-glucopyranoside* (**1**). Colorless gum; 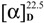 +4.35 (*c* 0.16, CH_3_OH); UV (CH_3_OH) *λ*_max_ (lg *ε*) 207 (4.82), 234 (4.26), 259 (4.21) nm; IR (film) *v*_max_ 3359, 2943, 1700, 1607, 1517, 1453,1369, 1277 cm^−1^; ^1^H and ^13^C-NMR data, see Table 1; positive HRESIMS *m/z* 679.1985 [M + Na]^+^ (calcd for C_33_H_36_O_14_Na, 679.2003).

**Table 1:** ^1^H- (400 MHz) and ^13^C-NMR (100 MHz) data of compound **1** in CD_3_OD (*δ* in ppm).

Position	*δ*_H_ (*J* in Hz)	*δ*_C_		Position	*δ*_H_ (*J* in Hz)	*δ*_C_
1		128.9, s		8′*α*	3.40, ddd (8.0, 6.0, 5.1)	60.4, d
2	7.03, d (1.7)	113.6, d		9′*α*	4.46, dd (9.2, 8.0)	72.4, t
3		149.4, s		9′*β*	3.76, dd (9.2, 6.0)	
4		147.5, s		-OCH_3_	3.84, s	56.7, q
5	6.72, d (8.1)	115.5, d			3.82, s	56.6, q
6	6.83 (overlapped)	122.1, d		G1′′	4.38, d (7.7)	100.2, d
7*β*	4.66, s	89.5, d		G2′′	3.04, m	74.9, d
8		99.3, s		G3′′	3.16, m	78.3, d
9*α*	4.38, d (10.4)	73.6, t		G4′′	3.16, m	72.0, d
9*β*	3.85, d (10.4)			G5′′	3.23, dd (15.2, 7.6)	75.7, d
1′		133.2, s		G6′′a	4.52, dd (11.8,1.8)	65.1, t
2′	7.01, d (1.7)	111.0, d		G6′′b	4.20, dd (11.8, 7.5)	
3′		148.5, s		1′′′		122.2, s
4′		147.4, s		2′′′, 6′′′	7.88, d (8.8)	133.0, d
5′	6.77, d (8.1)	116.3, d		3′′′, 5′′′	6.83, d (8.8)	116.4, d
6′	6.83 (overlapped)	120.2, d		4′′′		163.8, s
7′*β*	4.77, d (5.1)	87.2, d		7′′′		167.9, s

### 3.4. Hydrolysis of Compound 1 and Determination of the Absolute Configuration of the Sugar Moiety

Compound **1** (5 mg) was dissolved in MeOH (1 mL) and saturated aqueous Na_2_CO_3_ solution (4 mL) at room temperature for 4 h. The reaction mixture was concentrated under reduced pressure to provide crude product, which was purified by semi-preparative HPLC to obtain *p*-hydroxybenzoic acid and compound **2 **(3.2 mg). The later and cellulase (10 mg) were stirred in water (5 mL) at 37 °C for 3 days and extracted with EtOAc (3 mL × 3) [10]. The aglycone (1.4 mg), namely compound **3**, was purified from the organic phase. Part of the sugar residue, obtained from water layer, was analyzed by co-TLC with an authentic sample (CHCl_3_–MeOH–H_2_O, 6:4:1). The other sugar (0.8 mg) and L-cysteine methyl ester hydrochloride (2.0 mg) were dissolved in dry pyridine (1 mL) and then kept at 60 °C for 3 h. The reaction mixture was concentrated to dryness and then trimethylsilylimidazole was added to the residue, followed by stirring at 60 °C for 1 h [11]. Finally, the resultant solution was partitioned with n-hexane and water, and then the organic phase was subjected to GC analysis by using a HP-5MS capillary column (30 m × 0.25 mm × 0.25 µm, Agilent); column temperature, 200 °C; injection temp, 250 °C; detector FID, detector temp, 250 °C. A peak was found at the retention time of 18.10 min for compound **1**. When the standard solutions were prepared by the same reaction from corresponding standard glucose, the retention times of presilylated d-glucose and l-glucose were 18.12 and 18.72 min, respectively. Therefore, the sugar in compound **1** was determined to be d-glucose.

## 4. Conclusions

Phytochemical investigation on the whole plants of *Salvia scapiformis* Hance resulted in the isolation of one new compound **1** and seven known compounds **2**–**8**. Their structures were identified to be (+)-8-hydroxypinoresinol-8*α*-*O*-[6′′-*O*-(4′′′-hydroxybenzoyl)]-*β*-d-glucopyranoside (**1**), (+)-8*α*-hydroxypinoresinol-8-*O*-*β*-d-glucopyranoside (**2**), (+)-8*α*-hydroxypinoresinol (**3**), (+)-pinoresinol (**4**), (+)-medioresinol (**5**), ursolic acid (**6**), 4-epi-niga-ichigoside F1 (**7**), and niga-ichigoside F1 (**8**), respectively, on the basis of extensive spectroscopic analyses, chemical method, and comparison with spectroscopic data in the literature.

## Supplementary Materials

Supplementary materials can be accessed at: http://www.mdpi.com/1420-3049/18/9/11377/s1.
