# Supplementary material for: A New Lignan Glucoside from the Whole Plants of Salvia Scapiformis
Source: Molecules. 2013 Sep 13;18(9):11377–83. doi: 10.3390/molecules180911377 (PMC6269659; doi:10.3390/molecules180911377)

Supplementary Materials

Figure S1. HRESIMS spectrum of compound **1**.

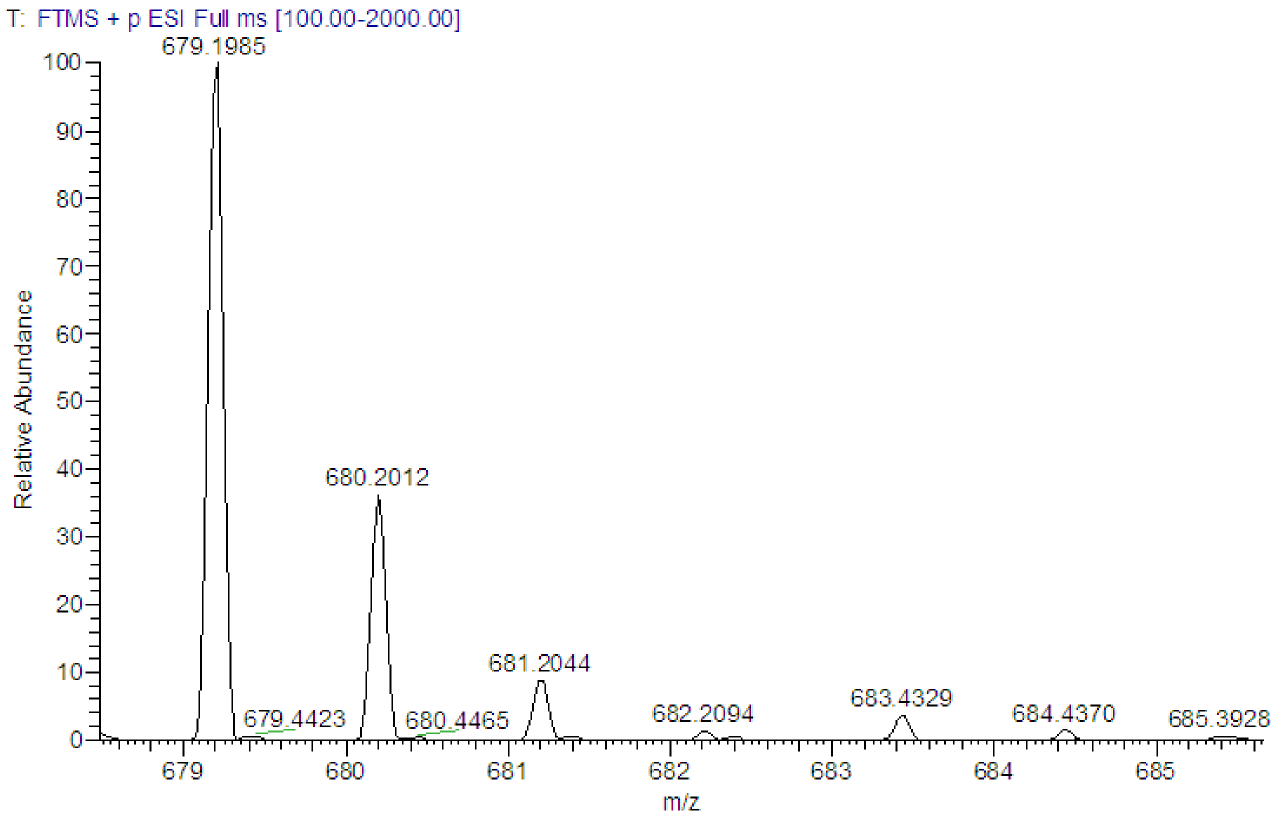

Figure S2. IR spectrum of compound **1.**

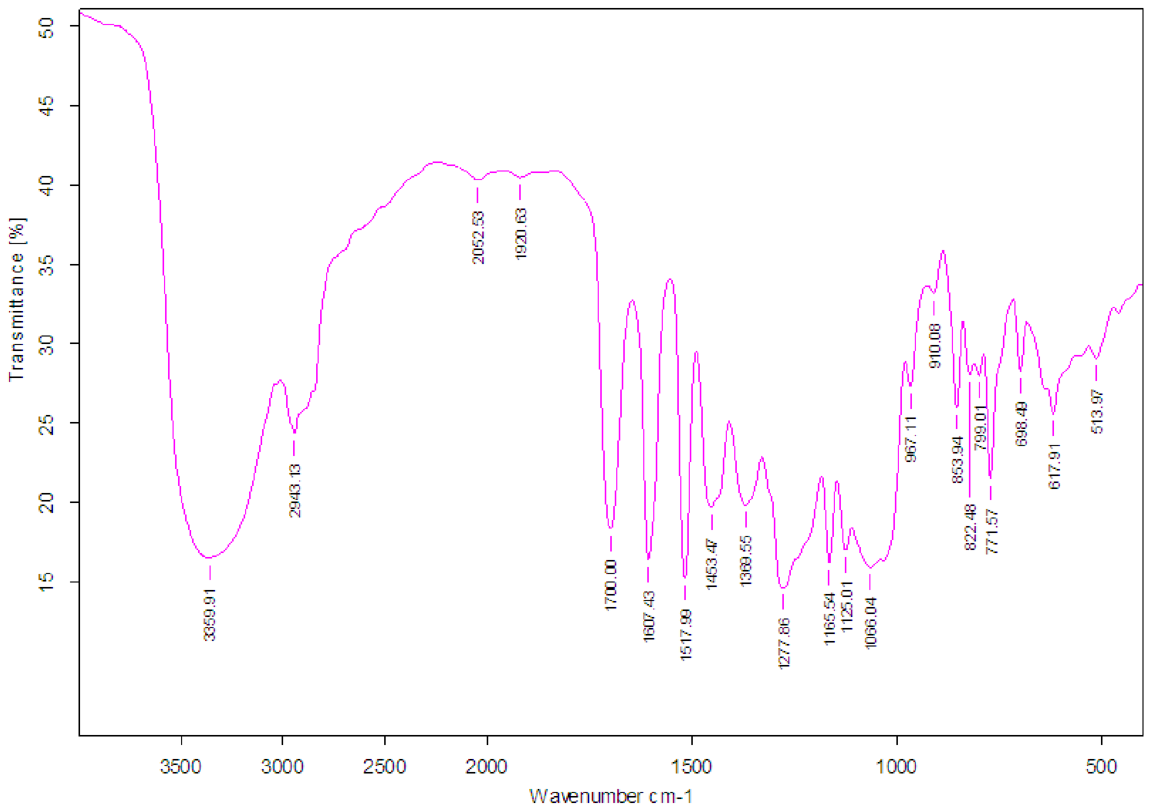

Figure S3. UV spectrum of compound **1.**

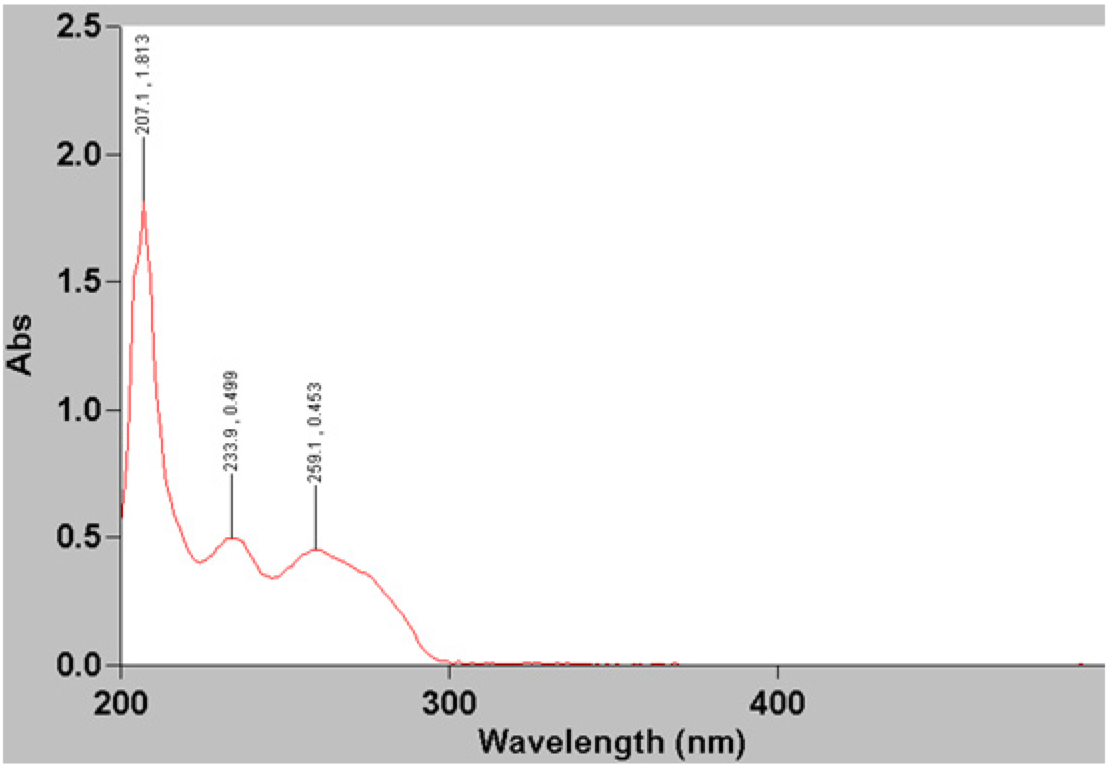

Figure S4. ^{1}H-NMR spectrum of compound **1.**

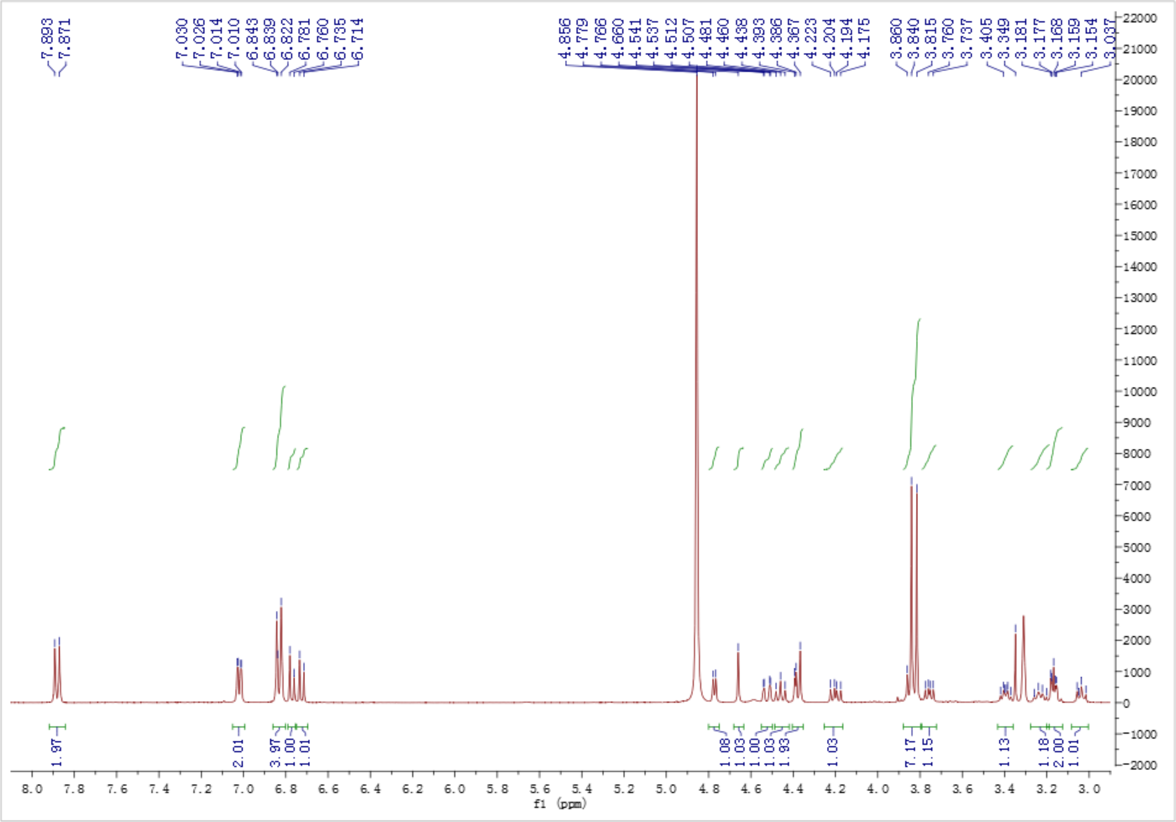

Figure S5. ^{13}C-NMR and DEPT spectra of compound **1.**

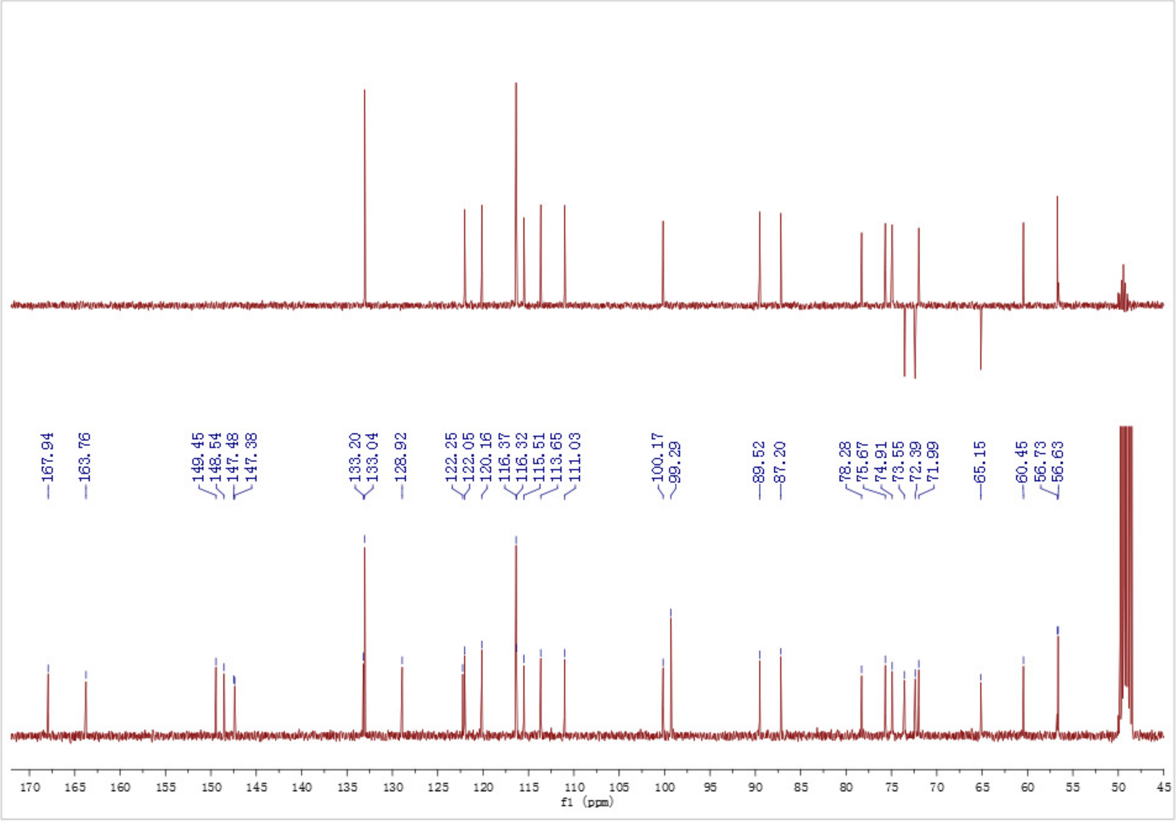

Figure S6. HSQC spectrum of compound **1.**

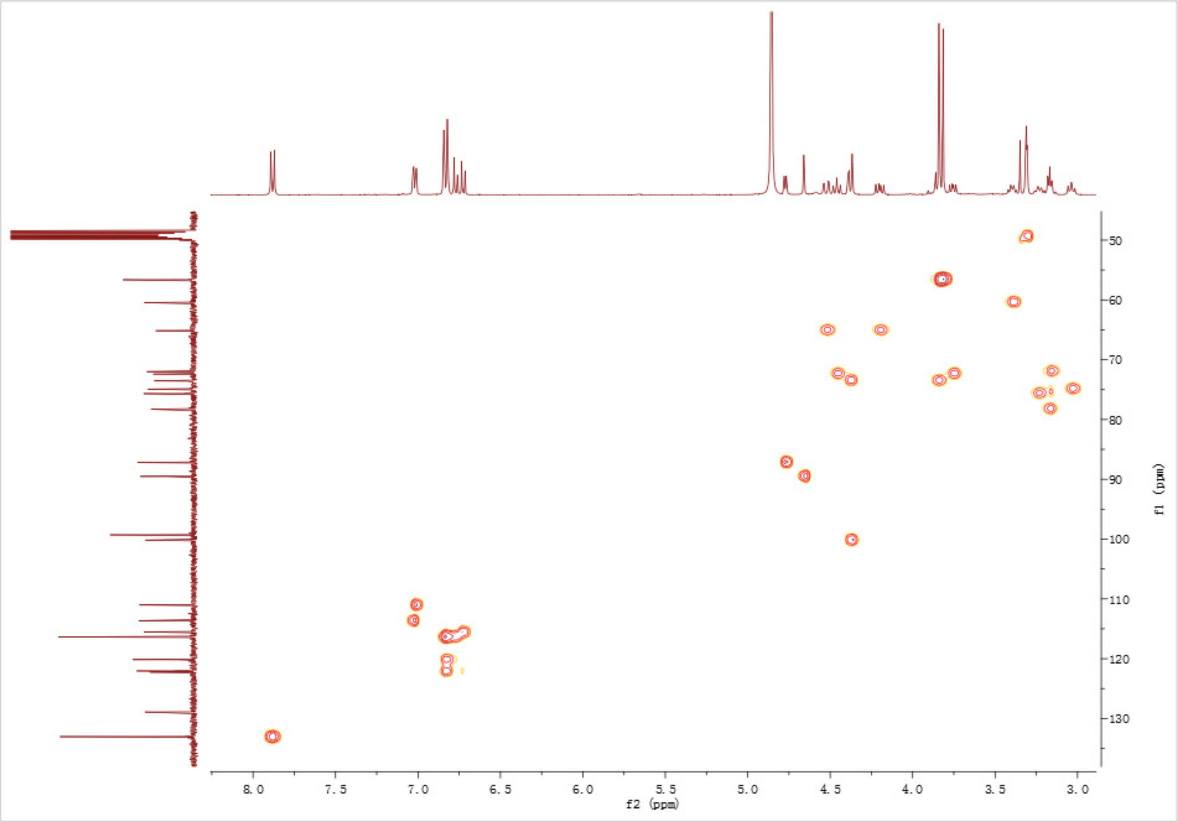

Figure S7. COSY spectrum of compound **1.**

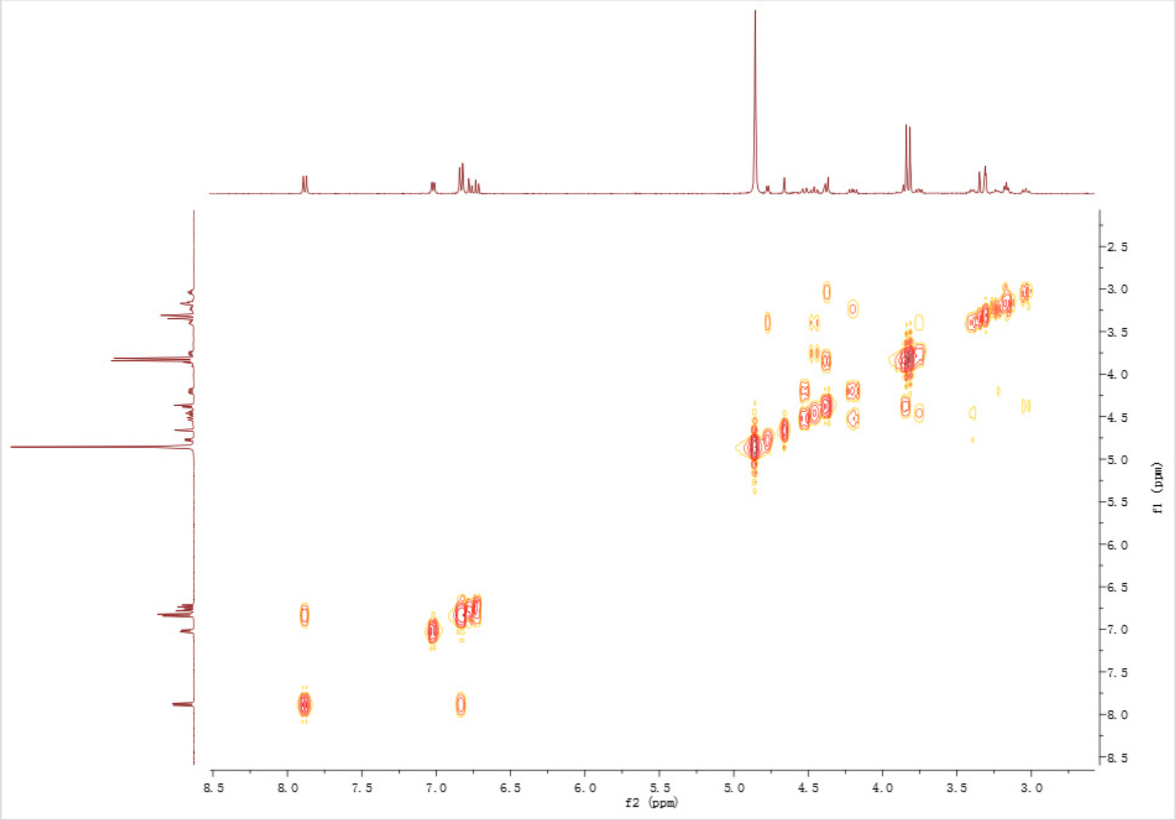

Figure S8. HMBC spectrum of compound **1.**

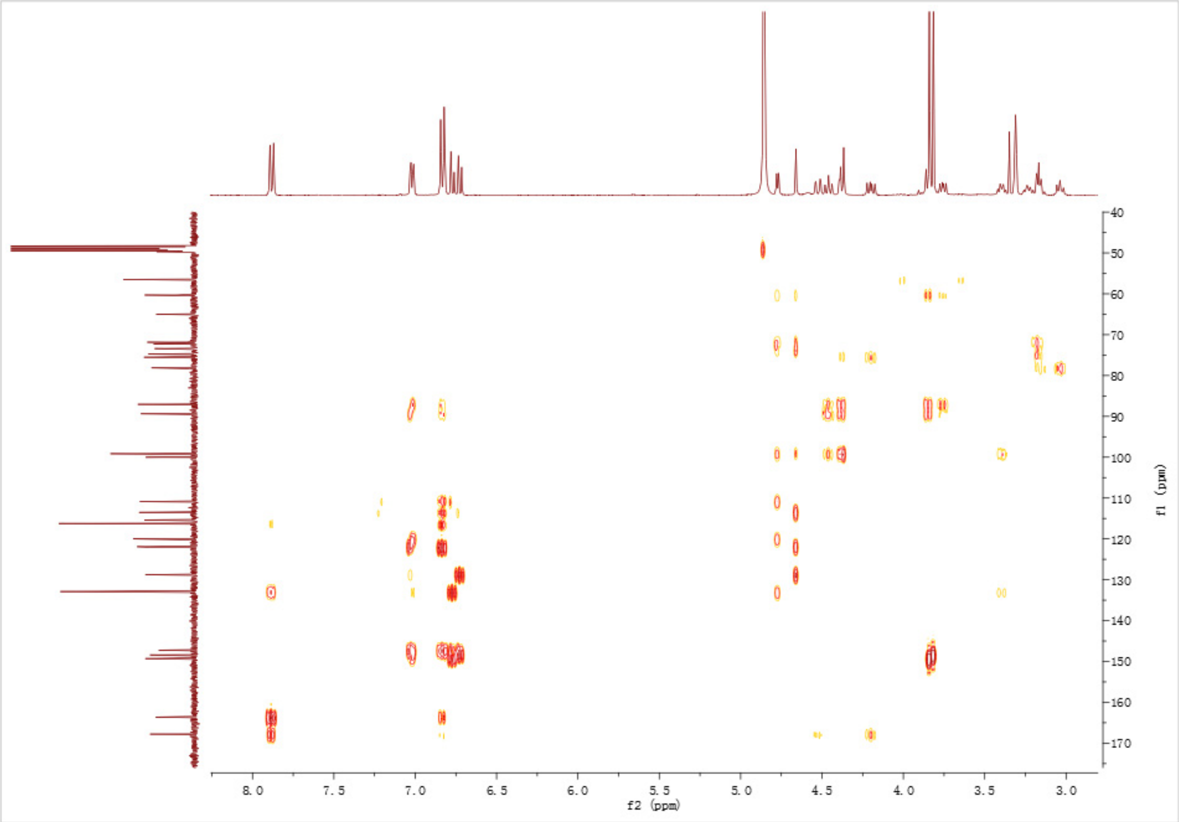

Figure S9. NOSEY spectrum of compound **1.**

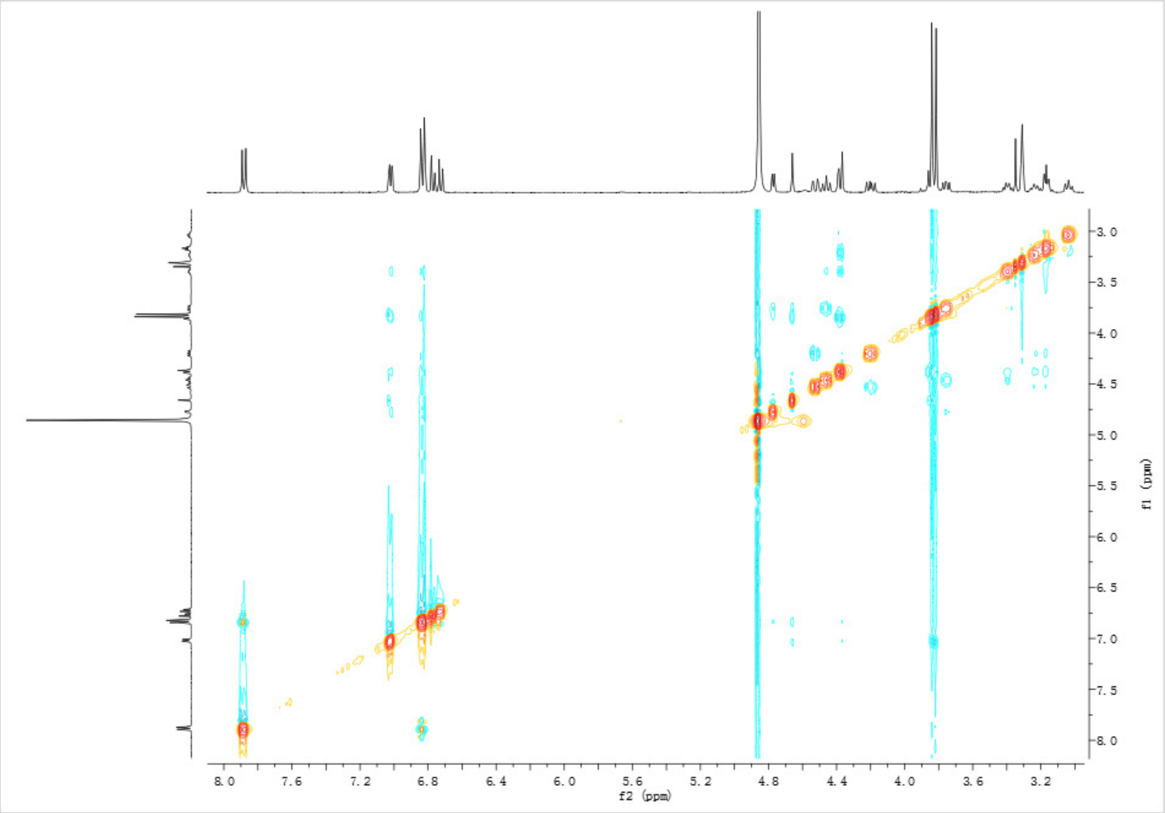

Figure S10. GS analysis of the sugar of compound **1** (**A**: L-glucose; **B**: D-Glucose; **C**: sugar of compound **1**).

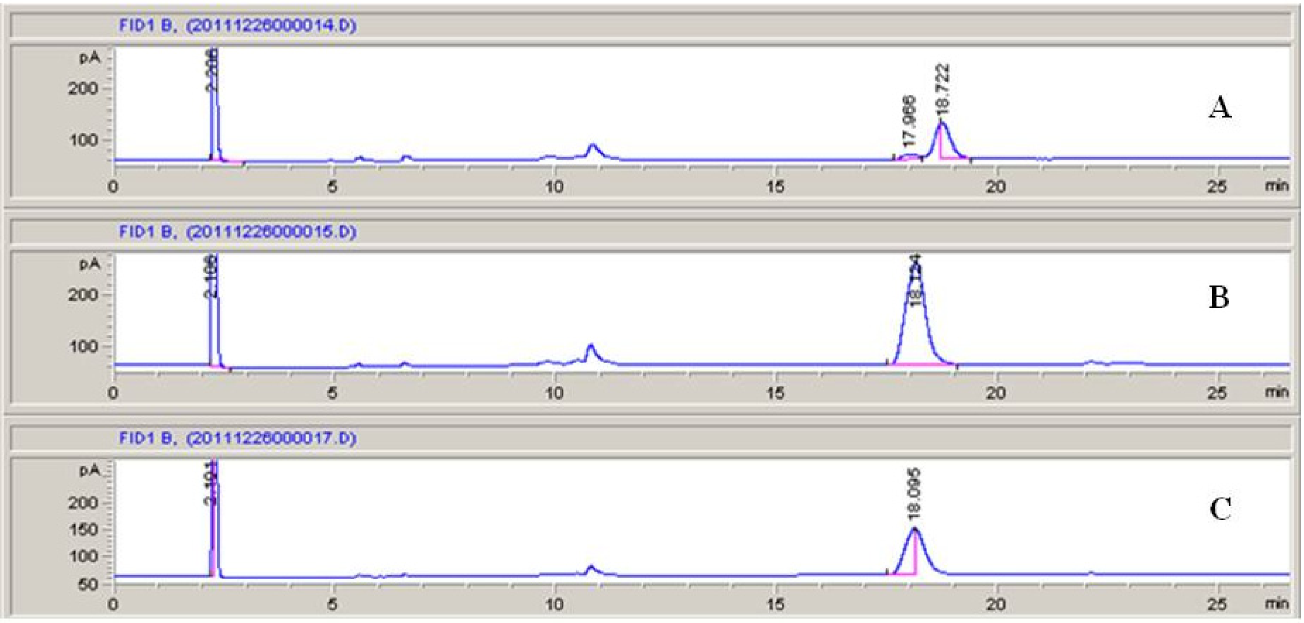

Supplement: Supplementary file 1 [file molecules-18-11377-s001.pdf]
